# Proof-of-Principle for Immune Control of Global HIV-1 Reactivation In Vivo

**DOI:** 10.1093/cid/civ219

**Published:** 2015-03-16

**Authors:** Nicola M. G. Smith, Petra Mlcochova, Sarah A. Watters, Marlene M. I. Aasa-Chapman, Neil Rabin, Sally Moore, Simon G. Edwards, Jeremy A. Garson, Paul R. Grant, R. Bridget Ferns, Angela Kashuba, Neema P. Mayor, Jennifer Schellekens, Steven G. E. Marsh, Andrew J. McMichael, Alan S. Perelson, Deenan Pillay, Nilu Goonetilleke, Ravindra K. Gupta

**Affiliations:** 1 Nuffield Department of Medicine, University of Oxford; 2 Department of Infection, Division of Infection and Immunity, University College London; 3 University College London Hospitals National Health Service (NHS) Foundation Trust; 4 Mortimer Market Centre, Central and North West London NHS Foundation Trust, United Kingdom; 5 Division of Pharmacotherapy and Experimental Therapeutics, Eshelman School of Pharmacy, University of North Carolina at Chapel Hill; 6 Anthony Nolan Research Institute, Royal Free Hospital; 7 Cancer Institute, University College London, United Kingdom; 8 Los Alamos National Laboratory, New Mexico; 9 Africa Centre for Health and Population Sciences, University of KwaZulu Natal, South Africa; 10 Department of Microbiology & Immunology, University of North Carolina at Chapel Hill

**Keywords:** elite control, HIV, cure, CD8, myeloablation

## Abstract

***Background.*** Emerging data relating to human immunodeficiency virus type 1 (HIV-1) cure suggest that vaccination to stimulate the host immune response, particularly cytotoxic cells, may be critical to clearing of reactivated HIV-1–infected cells. However, evidence for this approach in humans is lacking, and parameters required for a vaccine are unknown because opportunities to study HIV-1 reactivation are rare.

***Methods.*** We present observations from a HIV-1 elite controller, not treated with combination antiretroviral therapy, who experienced viral reactivation following treatment for myeloma with melphalan and autologous stem cell transplantation. Mathematical modeling was performed using a standard viral dynamic model. Enzyme-linked immunospot, intracellular cytokine staining, and tetramer staining were performed on peripheral blood mononuclear cells; in vitro CD8 T-cell–mediated control of virion production by autologous CD4 T cells was quantified; and neutralizing antibody titers were measured.

***Results.*** Viral rebound was measured at 28 000 copies/mL on day 13 post-transplant before rapid decay to <50 copies/mL in 2 distinct phases with t_1/2_ of 0.71 days and 4.1 days. These kinetics were consistent with an expansion of cytotoxic effector cells and killing of productively infected CD4 T cells. Following transplantation, innate immune cells, including natural killer cells, recovered with virus rebound. However, most striking was the expansion of highly functional HIV-1–specific cytotoxic CD8 T cells, at numbers consistent with those applied in modeling, as virus control was regained.

***Conclusions.*** These observations provide evidence that the human immune response is capable of controlling coordinated global HIV-1 reactivation, remarkably with potency equivalent to combination antiretroviral therapy. These data will inform design of vaccines for use in HIV-1 curative interventions.

## Abstract (precis)

It is unclear whether the human immune response is sufficiently potent to clear human immunodeficiency virus (HIV) type 1 latently infected cells globally reactivated by drug treatment. We report an elite controller who, following myeloablation and full HIV reactivation, achieved sustained control of viremia.

To date, the “Berlin patient” is the only known human immunodeficiency virus type 1 (HIV-1)–infected individual to achieve sustained nondetectable viremia without rebound following removal of combination antiretroviral therapy (cART). This success is likely due to a combination of transplantation with cells from a donor who lacked the chemokine (C-C motif) receptor 5 HIV-1 coreceptor that limited HIV-1 propagation, total body irradiation that depleted existing latently infected cells, and graft vs host disease. These observations have sparked the search for novel approaches to clear HIV reservoirs that reside largely in resting memory CD4+ T cells.

Recently, Shan et al, using an in vitro model of HIV latency, observed that HIV-1 reactivation was not itself adequate to destroy infected cells but that coculture with antigen-stimulated CD8 T cells could clear reactivated cells. This suggests that in addition to strategies that reactivate HIV latently infected CD4 T cells, future cure approaches may also need to stimulate immune responses, for example, through vaccination [1].

The host immune response plays a clear role in maintenance of virus load (VL) control in HIV-1 infection. The clearest example of this can be found in HIV-1–infected individuals who maintain VL <50 copies/mL without receiving cART (approximately 0.3% of HIV-1–infected individuals). These individuals, termed elite controllers (ECs), control acute HIV-1 viremia within weeks to months of infection [2], have higher CD4 T-cell counts than non-ECs, and progress far more slowly to AIDS [3]. CD8 T cells have been implicated in HIV-1 control in ECs largely because of enrichment of certain major histocompatibility complex class I alleles (human leucocyte antigen [HLA]-B*57, HLA-B*27, HLA-B*81) that are also associated with lower VLs and slower disease progression [4–8]. Protection in these individuals is thought to be largely mediated by the induction of HIV-1–specific CD8 T cells restricted predominantly by these HLA alleles. Mapping of these “protective” CD8 T-cell responses in ECs demonstrates that they typically target more conserved regions of HIV-1, commonly within the Gag protein. During chronic infection, HIV-1–reactive CD8 T-cell responses restricted by protective HLA are normally very strong or immunodominant within the individual. HIV mutation and escape from CD8 T-cell responses, when detected, have been associated with subsequent disease progression [9–11]. Further evidence of the role of CD8+ T cells comes from studies of macaques in which removal of anti–simian immunodeficiency virus (SIV) CD8+ T cells during acute infection and chronic infection resulted in loss of virus control, with control regained as CD8+ T cells returned [12–15].

Recent reports have highlighted the fact that elite virus control does not equate to HIV cure. ECs exhibit ongoing inflammation and innate immune activation, and have lower CD4 counts relative to uninfected individuals [16]. These symptoms are, in part, driven by residual levels of virus, with recent studies showing that cART can reduce inflammation in ECs [17]. This leaves the question of how well the immune response in chronically infected individuals, even in ECs, could respond to a coordinated global reactivation of the HIV reservoir proposed in HIV cure strategies.

In this study, we report a HIV-1–infected, antiretroviral therapy (ART)-naive EC who was treated for refractory myeloma with myeloablation and an autologous stem cell transplant (ASCT). Shortly after treatment, HIV-1 reactivation to 28 000 copies/mL was observed followed by rapid control of viremia to <50 copies/mL, remarkably at rates comparable to ART. This patient represents the first example of post-transplantation constraint of a large-scale virus reactivation mediated by the human immune response, presenting an unprecedented opportunity to measure both kinetics and in vitro correlates of efficacy.

## METHODS

### Total DNA Polymerase Chain Reaction

The duplexed polymerase chain reaction (PCR) was performed using 600 ng of patient template DNA with forward and reverse primer pairs for pyruvate dehydrogenase (PDH), PDH probe, long terminal repeat (LTR), and LTR probe. PCR was performed using Qiagen Multiplex Mastermix at 95°C for 15 minutes followed by 50 cycles at 94°C for 1 minute and 60°C for 1 minute. Assays were carried out in triplicate, and results were expressed as total HIV DNA copies per million cells.

### CD8 T-Cell Antiviral Assay (Extracellular p24)

The assay was performed as previously described [18].

### ELISpot for Interferon-γ Secreting Peripheral Blood Mononuclear Cell

T-cell epitopes were initially mapped against proteome-wide consensus clade C peptides (overlapping 15–18mers) in interferon-gamma (IFN-γ) ELISpot using peripheral blood mononuclear cells (PBMCs) collected at day 336. Peptides were then tested in triplicate at days 41 and 42 as previously described [19], and optimal epitopes were defined. Positive T-cell responses were 4 × background and >30 spot forming units/10^6^ cells following background subtraction [20].

### Intracellular Cytokine and Tetramer Staining

See Supplementary materials for details.

### Flow Cytometry of Lymphocyte Cells

See Supplementary materials for details.

#### Mathematical Modeling

A standard viral dynamic model [21] of HIV infection and effector cell responses was analyzed and represented as a system of differential equations (see Supplementary materials) that were then solved numerically using Berkeley Madonna v8.3.18.

## RESULTS

**Figure 1.**
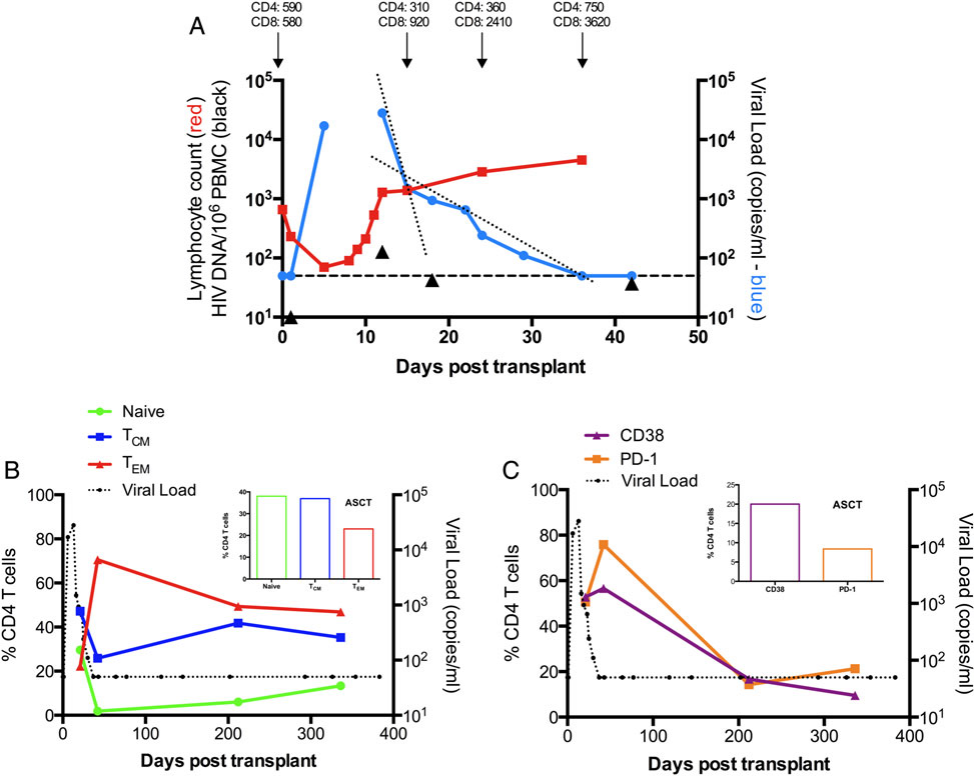
Dynamic changes in human immunodeficiency virus (HIV) viremia and CD4 T-cell activation levels following transplantation of CD34-enriched peripheral blood stem cells (day 0). *A*, Plasma HIV-1 RNA (copies/mL; blue circles), total lymphocyte count (×10^3^/mm^3^; red squares), and total viral DNA (HIV-1 copies/million peripheral blood mononuclear cells [PBMCs]; black triangles) over time following reinfusion of stem cells demonstrating first and second phase decay (dotted lines). Dashed line represents the lower limit of detection of the HIV-1 RNA viral load assay (50 copies/mL plasma). Note: The first HIV DNA measurement was below the limit of detection and has been plotted at 10 copies due to limitations of a logarithmic scale. Cytotoxic chemotherapy was administered at day 1. *B*, Frequency of CD4 naive and memory subsets over time: Tcm = central (CD45RO+CD27+) memory, Tem = effector (CD45RO+CD27−) memory (*C*). The frequency of CD38 (purple) and PD1 (orange) on CD4 T lymphocytes following stem cell infusion relative to plasma virus loads (black spheres). Inserts in (*B*) and (*C*) show cell subset frequencies in the pretreatment leukapheresis sample (autologous stem cell transplant [ASCT]).

A 59-year-old HIV-1 EC was treated for refractory myeloma using melphalan-induced myeloblation followed by an ASCT totaling 1.9 × 10^10^ cells. The ASCT contained both CD34 stem cells and mature lymphoid cells. The patient received etoposide–methylprednisolone–cytarabine–cisplatin conditioning and granulocyte colony-stimulating factor (CSF) to mobilize stem cells to the periphery. ART was not given in view of undetectable VL and concerns regarding drug toxicity. The melphalan treatment precipitated neutropenia and lymphopenia at +6 days (Supplementary Figure 1), at which time the HIV-1 VL had rebounded to 17 000 copies/mL (Figure 1*A*). At +13 days the lymphocyte count had increased to 1280 cells/mm^3^ and VL was 28 000 copies/mL (Figure 1*A*). The post-transplant environment contains high levels of interleukin-7 (IL-7) and IL-15, known to support homeostatic proliferation of memory, largely effector T cells, and may promote reactivation of HIV-1 from latently infected cells [22]. Consistently, CD4 T cells in this patient in the weeks following reactivation (day +21, day +42) exhibited a largely effector phenotype (Figure 1*B*). Cells showed elevated activation, as measured by CD38 and programmed death 1 (PD1) staining and increased Ki67 staining (Figure 1*C*, Table 1), without change in cell surface CD4 levels (data not shown), providing conditions conducive to HIV-1 reactivation and propagation.

**Table 1.** Changes in Lymphocyte Populations From Autologous Stem Cell Transplant to Resolution of Viremia

	Days Post-Transplant			
	−41	21	42	472
**Viral load**	<50	940	<50	<50
**Percentage of live lymphocytes**				
NK cells (CD56+)^a^	0.18	1.51	1.58	2.29
Of which CD16+CD56bright	ND	0.68	2.69	2.20
Of which CD16+CD56dim	ND	77.7	84.8	81.4
Of which CD16−CD56bright	ND	1.02	2.28	4.00
Of which CD16−CD56dim	ND	22.6	11.2	0.00
NKT-like cells (CD3+CD56+)	2.30	3.27	2.01	1.59
CD3 T cells (CD3+CD56−)	92.8	86.4	96.0	88.6
CD8 T cells (CD3+CD8+)	23.4	69.1	74.1	46.5
B cells (CD3−CD19+)	0.00	0.06	0.12	2.25
**Proliferation: % Ki67+**				
NK cells	92.5	92.1	81.5	10.5
NKT cells	58.3	63.0	75.3	3.20
CD8+ T cells	100	99.7	99.8	1.62
CD4+ T cells (CD3+CD8−CD56−)	90.9	90.5	99.0	1.77
B cells	ND	50.0	39.5	4.24

Lymphocyte populations were assessed by flow cytometry and are shown as a percentage of the total live cells (as determined by an amine-reactive viability dye) present in each sample. Autologous stem cells for day −41 or peripheral blood mononuclear cell for days 21,42, and 472.

Abbreviations: ND, not determined due to too few cells in the parent gate for analysis; NK, natural killer.

^a^ Defining markers for each population are shown in parentheses.

The VL rebound doubling time was 0.5 days, similar to that observed in acute infection (0.65 days) [23]. Importantly, the observation confirms that this EC harbored a fully replication-competent virus. This was followed by a VL decay characterized by 2 phases (Figure 1*A*), the first with t_1/2_ of 0.7 days and the second with t_1/2_ of 4.1 days. This first rate was derived from 2 measurements and therefore is subject to greater measurement error. We therefore incorporated the intraassay variability of the clinical VL test (1.9 fold), as determined by replicate measurements against known standards in our calculations. This produced an estimate of first phase t_1/2_ of 0.49–1.27 days. Comparison of these calculated rates with those described following ART treatment showed that the average first phase t_1/2_ of 0.7 days was very similar to a published estimate for the most potent cART [24]. The second phase t_1/2_ of 4.1 days was significantly shorter than previous published estimates of 14.25 (99% confidence interval, 4.780–23.370), derived using less potent antiretroviral combinations [25]. The measured peak in plasma VL RNA was accompanied by a rise in total HIV-1 DNA levels in PBMC from <10 copies/10^6^ cells pre-melphalan treatment to 127 copies/10^6^ cells at day +13, before subsequent decay with a t_1/2_ of 3.8 days (black triangles, Figure 1*A*).

By day +37, the VL had declined to undetectable levels. During the observed VL decline (day +13 to day +37), CD4 T-cell counts were >300 cells/mm^3^ (Figure 1*A*) compared with a total lymphocyte count of 70 cells/ mm^3^at day +6. Therefore, limited target cell availability is unlikely to have accounted for the VL decline. These cell kinetics were consistent with review of the terminal half-lives of the patient's pre-transplant conditioning regimens that suggested that the drugs received were unlikely to have impacted immune cell viability and function in the post-transplant environment (Supplementary text I). The patient achieved a favorable response to myeloma treatment and was in a stable plateau phase at +472 days.

The ASCT, when compared with the subsequent stage of durable virus control and normal ranges of lymphocyte subsets (data not shown), had diminished numbers of B, natural killer (NK), and NKT cells whereas T-cell numbers were overrepresented, consistent with the effects of CSFs prior to the harvest (Table 1) [22]. The kinetics of lymphocyte populations during virus rebound were also consistent with those of immune recovery post-transplantation including HIV-1–infected patients [22, 26]. This included increases in frequencies of NK subsets within weeks of transplant as well as a striking expansion of CD8+ T cells (Table 1). CD19+ B cells did not recover until after virus control had been regained (Table 1). Neutralizing antibody titers were modest (Supplementary Table 1) in comparison to titers commonly seen in chronic HIV-1 infection of viremic patients and individuals undergoing ART interruption [27, 28] and therefore are unlikely to have contributed to the control observed.

**Figure 2.**
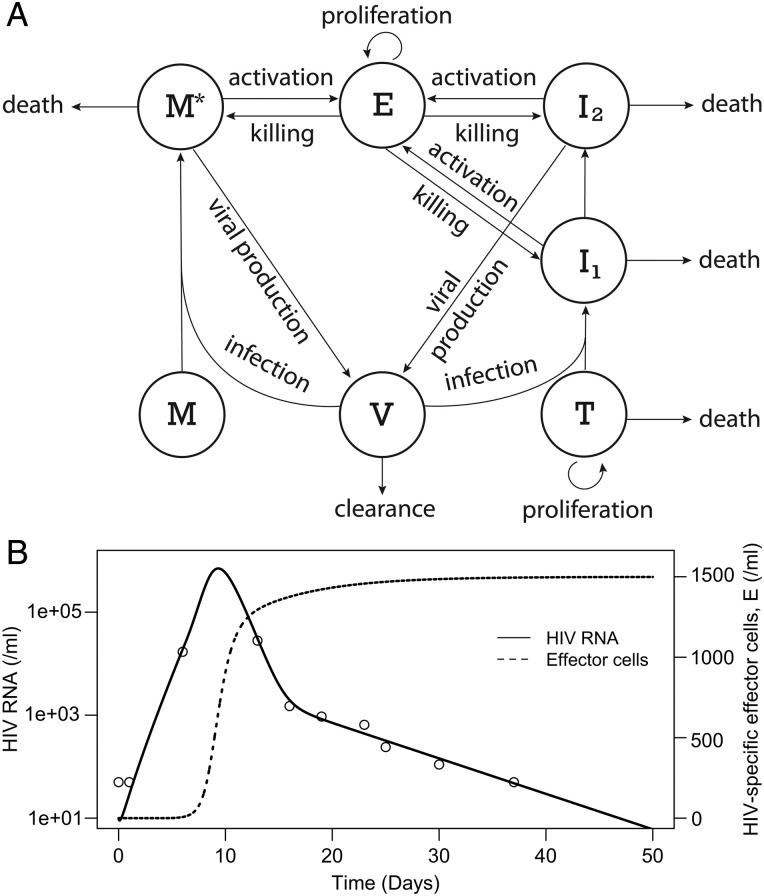
Mathematical modeling is consistent with CD8+ T-cell–mediated killing of infected cells. *A*, Schematic illustration of mathematical model. On the left, cells *M*, which might be macrophages or resting CD4+ T cells, when infected by human immunodeficiency virus type 1 (HIV-1), *V*, become long-lived infected cells, M*, which are estimated to be responsible for a few percent of body-wide viral production [25]. The cells on the right, *T*, are the major targets of HIV infection. After infection, the cells, *I_1_*, are in an eclipse phase and do not produce virus until they transition into productively infected cells, *I_2_*. Both *I_2_* and *M* cells produce virus, *V*. This free virus can, in turn, infect uninfected target cells, *T* and *M*. When effector cells, *E*, contact infected cells they become activated, resulting in both killing of the infected target and proliferation of the effector cell. The model also considers the death rates of cells and the viral clearance rate. *B*, Results of simulating the model given by Eq. (1) detailed in the Supplementary text; HIV-1 RNA/mL (solid) and effector cells/ mL (dotted). Parameters used are as follows: *r* = 0.8 d^−1^, β = 1.66 × 10^−8^ mL d^−1^, β*_M_* = 4.14 × 10^−9^ mL d^−1^, *d_T_* = 0.5 d^−1^, *T_max_* = 10^6^/mL, t_1_ = 6 d, *M* = 6 × 10^4^/mL, δ = 0.9 d^−1^, δ*_E1_* = 0.01 mL d^−1^, δ*_E2_* = 0.001 mL d^−1^, δ_EM*_ = 10^−4^ mL d^−1^, δ*_M_* = 0.01 d^−1^, *k* = 1.0 d^−1^, *q* = 0.2 d^−1^, *K* = 1, *p* = 20 000 d^−1^, *p_M_* = 200 d^−1^, and *c* = 23 d^−1^. The initial conditions were *T*(0) = 6 × 10^5^ /mL, *I_1_*(0) = *I_2_*(0) = M*(0) = 0, *E*(0) = 0.05/mL, and *V*(0) = 10/mL. The viral loads generated by the model agree with the ones measured in the patient (open circles). The magnitude of the effector cell response is also consistent in magnitude to the measured level of HIV-1–specific CD8 T-cell responses at day 42 shown in Figure 3.

We used mathematical modeling [21] to determine whether the patient's unique HIV-1 viral kinetics and cell death rates were consistent with established models of cytolytic immune cell viral control. A standard viral dynamic model, shown schematically in Figure 2*A*, was analyzed in which infected cells in the eclipse phase, *I*_1_, transition to productive infection, *I*_2_, and with both populations as well as long-lived infected cells, M*, being potential targets of immune cell killing. In the model, cytolytic effectors, *E*, were stimulated by the presence of infected cells and grew with saturating kinetics. The model was represented as a system of differential equations and solved numerically (Supplementary material). The model was able to reproduce the VL changes observed in the patient (Figure 2*B*). Furthermore, by changing parameters, we were able to explore the effects of the different biological processes being modeled. We found that with parameters that resulted in a VL peak at about day 8, the effector cells that started at a low precursor frequency were still sufficient to expand rapidly near the peak VL and reproduce the viral load decline kinetics we observed (Figure 2*B*).

**Figure 3.**
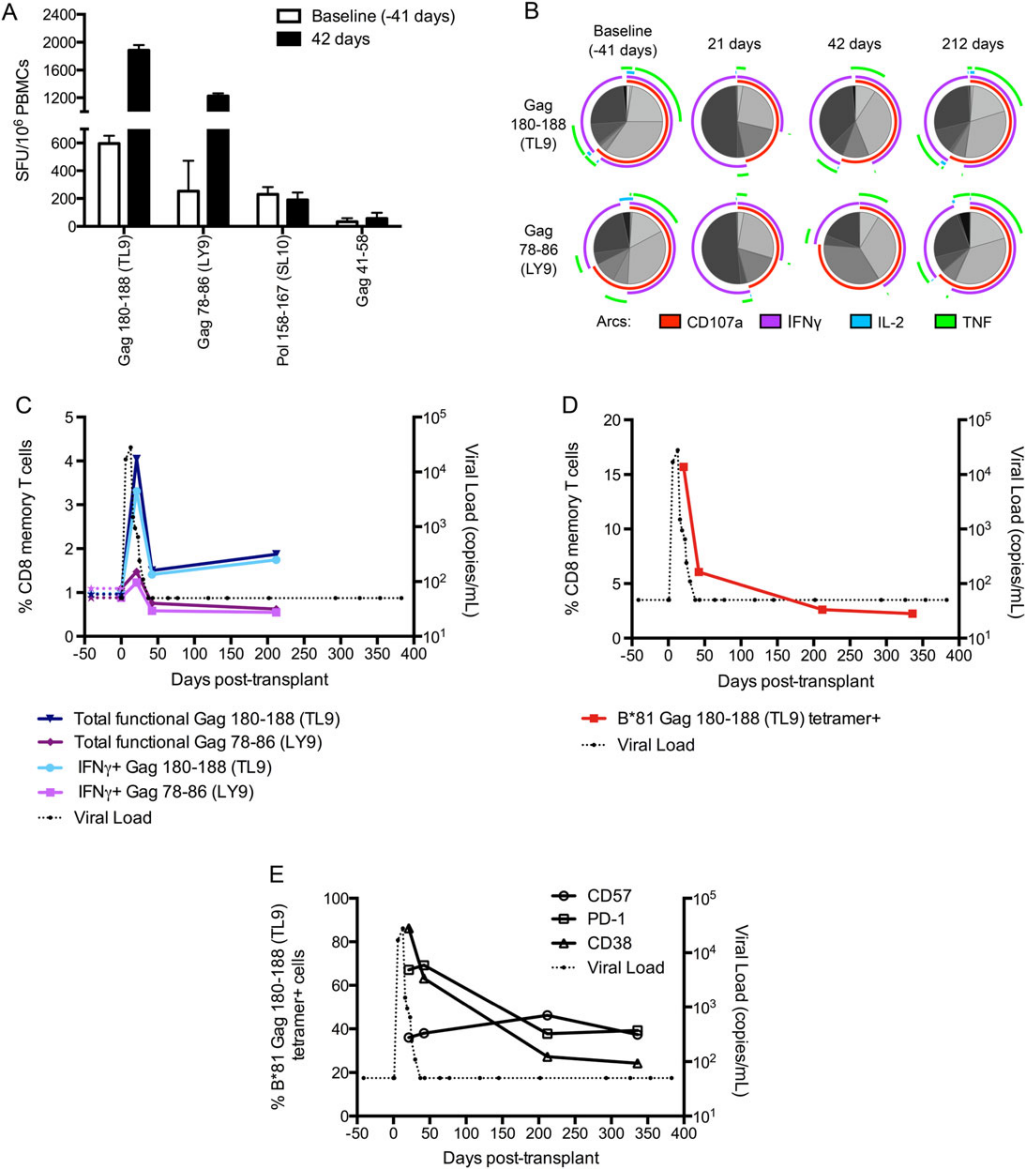
Human immunodeficiency virus type 1 (HIV-1)–specific CD8+ T-cell responses are highly functional, expanding strongly with control of HIV-1 reactivation. T-cell responses were initially mapped by interferon-gamma (IFN-γ) enzyme-linked immunospot at +336 days and were refined to optimal epitopes based on the patient's human leukocyte antigen type. *A*, IFN-γ responses to mapped peptides were assayed in the baseline sample and peripheral blood mononuclear cells (PBMCs) at +42 days post-transplantation. Results represent mean ± standard deviation spot forming units/10^6^ (SFU) cells of triplicate measurements. *B*, Functionality of TL9 and LY9 CD8 memory T-cell responses before (−41 days, viral load [VL] <50), during (+21 days, VL measured at +19 and +23 days was 940 and 650 copies/mL, respectively), and after (+42 and +212 days, VL < 50) virus rebound. Colored arcs indicate cytokines. Shaded sectors show the proportion of each cytokine combination. *C*, Gag 180–188 (TL9) and Gag 78–86 (LY9) T-cell responses measured by intracellular cytokine flow cytometry prior to and during VL rebound. Percentage of CD8+ memory T cells expressing 1 or more of IFN-γ, tumor necrosis factor (TNF)-alpha, interleukin-2 (IL-2), and CD107a, as well as IFN-γ only, shown. *D*, Kinetics of B*81:01 Gag180–188 tetramer+ cells and VL during the study period. *E*, Changes in expression of activation markers CD57, PD-1, and CD38 by tetramer+ CD8 memory T cells during VL decline. Note: PBMCs prior to chemotherapy were unavailable; however, a sample from the leukapheresis at day −41 that was transplanted at day 0 was considered to be a baseline and representative of HIV-1–specific T-cell responses prior to chemotherapy.

We determined whether HIV-1–specific CD8 T-cell responses were dominating the observed control. Although PBMCs prior to melphalan treatment were unavailable, we did have access to the ASCT taken 41 days before transplantation. We assumed that these cells were the baseline sample as they represented the major source of memory T cells at day 0. Mapping of HIV-1–specific T-cell responses identified epitopes in Gag and Pol detectable in the baseline and at subsequent time points (Figure 3*A*). Direct virus sequencing showed no amino acid changes in any of the Gag or Pol CD8 T-cell–restricted epitopes, indicating no virus escape from T cells during the study period (+472 days from transplantation; data not shown).

Focusing on the dominant HLA-B*81–restricted TL9 and LY9 Gag-specific responses, we examined IFN-γ, tumor necrosis factor-alpha (TNF-α), and IL-2 production and expression of CD107a+ lytic granules by intracellular cytokine flow cytometry before (−41 days), during (+21 days), and after (+42 and +212 days) viral rebound. Very similar functional patterns were observed for both epitopes: 65% of baseline specific T cells exhibited >1 function and almost 20% exhibited triple IFN-γ, TNF-α, and CD107a expression following stimulation (Figure 3*B*). These proportions are consistent with functional profiles of HIV viremic controllers who exhibit broader CD8 T-cell functionality than viremic progressors [7]. During virus rebound (day 21), the proportion of single positive, largely IFN-γ or CD107a positive, cells increased in both the TL9- and LY9-specific populations by around 3-fold. By day 212, the functional phenotype of both cell populations was very similar to that observed at baseline. Analysis of the total measured functional T-cell response (at least 1 function) found that the TL9- and LY9-specific cells accounted for 2% of total memory CD8+ T cells in the baseline sample (Figure 3*C*), equating to approximately 26 million cells transferred in the ASCT (details presented in the “Methods” section). Upon virus reactivation, the TL9-specific functional T-cell response increased >4-fold (Figure 3*C*), and parallel HLA-B*81-TL9 tetramer staining showed that almost 16% of circulating memory CD8 T cells were specific following rebound (Figure 3*D*). TL9 reactive cells exhibited a CD45RO+CD27+ central memory phenotype (data not shown), and CD38 and PD1 staining showed that cells were highly activated around peak virus rebound. In contrast, CD57 levels, which are a hallmark of extensive cellular division, were stable during virus rebound (Figure 3*E*). These T-cell frequencies (Figure 3*A*), when combined with the high, absolute CD8 T-cell counts measured post-HIV reactivation (Figure 1), were consistent with the “effector” numbers that yielded virus control in our modeling analysis (Figure 2*B*).

**Figure 4.**
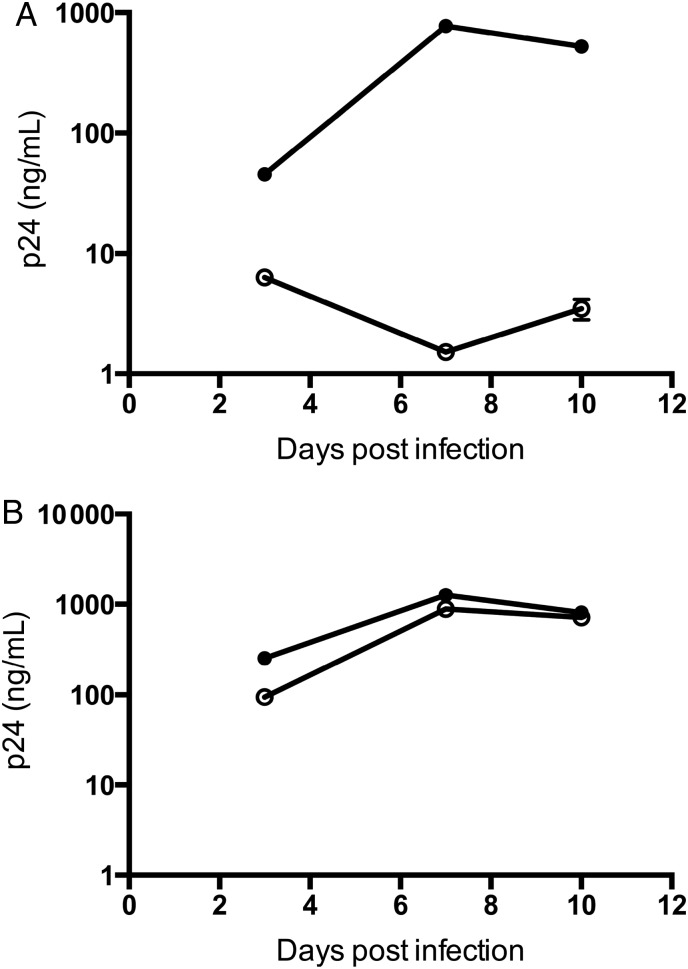
Autologous CD8+ T cells potently suppress extracellular human immunodeficiency virus type 1 (HIV-1). HIV-1 capsid p24 production was measured using enzyme-linked immunosorbent assay in culture supernatants (mean ± standard deviation, n = 3) from elite controller-derived CD4+ T cells (days 326, 354, and 383) infected with HIV-1 BaL (*A*), as well as CD4+ T cells derived from chronically HIV-1–infected individuals (n = 3) (*B*) in the absence (closed) or presence (open symbols) of autologous unstimulated CD8+ T cells (at ratio 1:1). Each experiment was performed in triplicate, and data shown are representative of at least 2 independent experiments.

We quantified the ability of the patient's CD8 T cells to limit virus production following infection of autologous T cells with an HIV laboratory isolate. Fresh CD8 T cells isolated following control of HIV-1 viremia (days +326 and +354) reduced HIV-1 p24 derived from autologous CD4 T cells by 3 orders of magnitude by day 7 in culture (Figure 4*A* and 4*B*). We then estimated the in vitro t_1/2_ for virus inhibition. When autologous CD4 cells were infected in vitro, supernatant p24 increased at a rate of 0.75/day. In contrast, in the presence of autologous CD8 T cells at a 1:1 ratio, p24 decreased at a rate of 0.75/day, translating to a t_1/2_ of 0.93 days, which is similar to the rates of VL decline observed in vivo (Figure 1*A*).

## DISCUSSION

Emerging data from laboratory studies relating to HIV-1 cure suggest that vaccination to stimulate the host immune response, particularly cytotoxic cells, may be critical to the clearing of reactivated HIV-1–infected cells following use of latency reversing agents (LRAs). However, evidence for this approach in humans is lacking, and parameters required for a vaccine are unknown because opportunities to study HIV-1 reactivation are extremely rare. We combined in vivo, in silico, and in vitro approaches to demonstrate for the first time that the human immune response is capable of controlling an iatrogenic large-scale HIV-1 reactivation, as might be induced by LRAs.

The data were derived from a HIV-1 seropositive EC who underwent myeloablation and ASCT as treatment for myeloma. The patient rapidly regained control of viral reactivation with kinetics comparable to those previously observed with ART. Our primary observation was the rapid rate of virus decline in the patient from day 13 to day 37 post-transplant. A biphasic decline was observed: the first slope, though derived from limited measurements, had a t_1/2_ of 0.71 (0.49–1.27) days and was equivalent to that observed in the most potent ART regimens. Notably, the higher estimate of 1.27 days was still classified as rapid in the absence of therapy and very similar to the estimated t_1/2_ of HIV-1 in post-peak virus decline in acute viremia [23]. The t_1/2_ in the second phase was 4.7 days, also comparable to observations in ART-treated patients.

In the absence of ART, what parameters produced these slopes in the patient? First, analysis of virus kinetics found that the virus upslope was more rapid than that observed in acute viremia [23]. This suggested that the rebounding virus in this EC was highly competent for replication in vivo*,* and therefore the virus itself could not explain the decline observed. A recent report also showed that virus isolates from ECs were fully replication-competent in vivo using a humanized mice model of HIV-1 infection [29]. Second, in the pre-ART era, HIV-1–infected noncontrollers undergoing similar myeloablation and transplantation procedures consistently exhibited high viremia post-transplant, indicating that the conditioning regimens do not, when used alone, constrain HIV-1 replication [30]. Further, analysis of pharmacokinetic data of the patient's conditioning regimens indicated that they were unlikely to impact cell viability, which is consistent with the patient's CD4 T cell count, which remained >300 cells/mm^3^ as VL control was regained. In summary, the VL kinetics in the patient are not explained by poor virus replication nor limiting target cell availability.

During this rebound period, the patient's HIV-1–specific CD8 T-cell response expanded strongly. This occurred in response to HIV-1 reactivation and was unlikely a by-product of homeostatic proliferation. In macaques, IL-15 treatment of durably suppressed SIV-infected monkeys similarly induced homeostatic proliferation of CD4 and CD8 effector T-cell subsets but did not increase SIV-specific T-cell responses [31]. T-cell mapping showed that in the patient, the immunodominant CD8 T-cell epitope corresponded to the dominant T-cell response observed in HLA-B*81:01 patients. This T-cell response has been detected in patients with acute HIV-1 infection, including those who subsequently achieved elite control. The target epitope is highly conserved at the population level, and subsequent escape is associated with significant loss of replicative fitness [20, 32]. We followed the kinetics of the dominant T-cell responses and found strong expansion in response to virus rebound, though without evidence that cells were terminally expanded. These cell populations rapidly declined following reacquisition of virus control. No virus escape through mutation of T-cell epitopes was observed during the period of study, consistent with T cells exerting an ongoing immune pressure. Analysis of other CD8 T-cell functions during the rebound period found that HIV-1–specific cells became highly activated and, interestingly, appeared to exhibit reduced oligofunctionality relative to HIV-1–specific cells analyzed at baseline. Activation levels rapidly subsided when virus control was regained, with broader functionality in terms of cytokine and lytic marker production again observed.

Last, strong in vitro suppression of HIV was observed at a subsequent time point following virus control (when HIV-1–specific CD8 T-cell frequencies were much lower). Importantly, the frequencies of the patient's HIV-1–specific T-cell responses, when combined with the high, absolute CD8 T-cell counts measured post-HIV reactivation, were consistent with the “effector” numbers in modeling that produced VL kinetics very similar to that observed in our patient. Together, these data suggest that the patient's HIV-1–specific CD8 T-cell response following reactivation directly contributed to the virus control observed. This hypothesis is further supported by recent studies of viremic controller macaques in which virus rebound following CD8 depletion by antibodies and regain of virus control as CD8 T cells returned were observed [14, 15]. Additional support is garnered from the transplantation biology setting where cytomegalovirus (CMV) reactivation is often observed post-transplantation, with the level of CMV-reactive CD8 T cells correlating with control of CMV viremia [33].

However, as a single patient study, our observations are associative. We cannot quantify the precise in vivo contribution of CD8 T cells to the control observed and do not exclude a role for other subsets, particularly NK cells, in this patient, contributing either directly to anti–HIV-1 cytotoxicity or indirectly by conferring a clinical benefit against the patient's myeloma [34]. The T-cell responses induced in this patient were typical of those induced in other ECs, suggesting that vaccination regimens that induce broadly functional CD8 T-cell responses, with good replicative potential and targeting conserved regions of HIV-1, might be more effective at recognizing and clearing reactivated HIV-infected cells. However, challenges to this approach still remain, for example, the requirement for immune-boosting strategies that target conserved, unmutated epitopes following the recent finding that HIV-1 proviruses in latently infected cells from chronically infected patients almost always contain cytotoxic T lymphocyte escape mutations [35].

## Supplementary Data

Supplementary materials are available at *Clinical Infectious Diseases* online (http://cid.oxfordjournals.org). Supplementary materials consist of data provided by the author that are published to benefit the reader. The posted materials are not copyedited. The contents of all supplementary data are the sole responsibility of the authors. Questions or messages regarding errors should be addressed to the author.

Supplementary Data

## References

[1] Shan L Deng K Shroff NS Stimulation of HIV-1-specific cytolytic T lymphocytes facilitates elimination of latent viral reservoir after virus reactivation. Immunity 2012; 36:491–501. 22406268 10.1016/j.immuni.2012.01.014 PMC3501645

[2] Goujard C Chaix ML Lambotte O Spontaneous control of viral replication during primary HIV infection: when is “HIV controller” status established? Clin Infect Dis 2009; 49:982–6. 19681706 10.1086/605504

[3] Deeks SG Walker BD Human immunodeficiency virus controllers: mechanisms of durable virus control in the absence of antiretroviral therapy. Immunity 2007; 27:406–16. 17892849 10.1016/j.immuni.2007.08.010

[4] Migueles SA Sabbaghian MS Shupert WL HLA B*5701 is highly associated with restriction of virus replication in a subgroup of HIV-infected long term nonprogressors. Proc Natl Acad Sci U S A 2000; 97:2709–14. 10694578 10.1073/pnas.050567397 PMC15994

[5] Emu B Sinclair E Hatano H HLA class I-restricted T-cell responses may contribute to the control of human immunodeficiency virus infection, but such responses are not always necessary for long-term virus control. J Virol 2008; 82:5398–407. 18353945 10.1128/JVI.02176-07 PMC2395228

[6] Kiepiela P Leslie AJ Honeyborne I Dominant influence of HLA-B in mediating the potential co-evolution of HIV and HLA. Nature 2004; 432:769–75. 15592417 10.1038/nature03113

[7] Betts MR Nason MC West SM HIV nonprogressors preferentially maintain highly functional HIV-specific CD8+ T cells. Blood 2006; 107:4781–9. 16467198 10.1182/blood-2005-12-4818 PMC1895811

[8] Lambotte O Boufassa F Madec Y HIV controllers: a homogeneous group of HIV-1-infected patients with spontaneous control of viral replication. Clin Infect Dis 2005; 41:1053–6. 16142675 10.1086/433188

[9] Kelleher AD Long C Holmes EC Clustered mutations in HIV-1 gag are consistently required for escape from HLA-B27-restricted cytotoxic T lymphocyte responses. J Exp Med 2001; 193:375–86. 11157057 10.1084/jem.193.3.375 PMC2195921

[10] Goulder PJ Phillips RE Colbert RA Late escape from an immunodominant cytotoxic T-lymphocyte response associated with progression to AIDS. Nat Med 1997; 3:212–7. 9018241 10.1038/nm0297-212

[11] Barouch DH Kunstman J Kuroda MJ Eventual AIDS vaccine failure in a rhesus monkey by viral escape from cytotoxic T lymphocytes. Nature 2002; 415:335–9. 11797012 10.1038/415335a

[12] Jin X Bauer DE Tuttleton SE Dramatic rise in plasma viremia after CD8(+) T cell depletion in simian immunodeficiency virus-infected macaques. J Exp Med 1999; 189:991–8. 10075982 10.1084/jem.189.6.991 PMC2193038

[13] Schmitz JE Kuroda MJ Santra S Control of viremia in simian immunodeficiency virus infection by CD8+ lymphocytes. Science 1999; 283:857–60. 9933172 10.1126/science.283.5403.857

[14] Pandrea I Gaufin T Gautam R Functional cure of SIVagm infection in rhesus macaques results in complete recovery of CD4+ T cells and is reverted by CD8+ cell depletion. PLoS Pathog 2011; 7:e1002170. 21829366 10.1371/journal.ppat.1002170 PMC3150280

[15] Friedrich TC Valentine LE Yant LJ Subdominant CD8+ T-cell responses are involved in durable control of AIDS virus replication. J Virol 2007; 81:3465–76. 17251286 10.1128/JVI.02392-06 PMC1866056

[16] Krishnan S Wilson EM Sheikh V Evidence for innate immune system activation in HIV type 1-infected elite controllers. J Infect Dis 2014; 209:931–9. 24185941 10.1093/infdis/jit581 PMC3935475

[17] Hatano H Yukl SA Ferre AL Prospective antiretroviral treatment of asymptomatic, HIV-1 infected controllers. PLoS Pathog 2013; 9:e1003691. 24130489 10.1371/journal.ppat.1003691 PMC3795031

[18] Saez-Cirion A Shin SY Versmisse P Barre-Sinoussi F Pancino G Ex vivo T cell-based HIV suppression assay to evaluate HIV-specific CD8+ T-cell responses. Nat Protoc 2010; 5:1033–41. 20539279 10.1038/nprot.2010.73

[19] Goonetilleke N Moore S Dally L Induction of multifunctional human immunodeficiency virus type 1 (HIV-1)-specific T cells capable of proliferation in healthy subjects by using a prime-boost regimen of DNA- and modified vaccinia virus Ankara-vectored vaccines expressing HIV-1 Gag coupled to CD8+ T-cell epitopes. J Virol 2006; 80:4717–28. 16641265 10.1128/JVI.80.10.4717-4728.2006 PMC1472051

[20] Liu MK Hawkins N Ritchie AJ Vertical T cell immunodominance and epitope entropy determine HIV-1 escape. J Clin Invest 2013; 123:380–93. 23221345 10.1172/JCI65330 PMC3533301

[21] Perelson AS Ribeiro RM Modeling the within-host dynamics of HIV infection. BMC Biol 2013; 11:96. 24020860 10.1186/1741-7007-11-96 PMC3765939

[22] Williams KM Gress RE Immune reconstitution and implications for immunotherapy following haematopoietic stem cell transplantation. Best Pract Res Clin Haematol 2008; 21:579–96. 18790456 10.1016/j.beha.2008.06.003 PMC2577193

[23] Ribeiro RM Qin L Chavez LL Li D Self SG Perelson AS Estimation of the initial viral growth rate and basic reproductive number during acute HIV-1 infection. J Virol 2010; 84:6096–102. 20357090 10.1128/JVI.00127-10 PMC2876646

[24] Markowitz M Louie M Hurley A A novel antiviral intervention results in more accurate assessment of human immunodeficiency virus type 1 replication dynamics and T-cell decay in vivo. J Virol 2003; 77:5037–8. 12663814 10.1128/JVI.77.8.5037-5038.2003 PMC152136

[25] Perelson AS Essunger P Cao Y Decay characteristics of HIV-1-infected compartments during combination therapy. Nature 1997; 387:188–91. 9144290 10.1038/387188a0

[26] Simonelli C Zanussi S Pratesi C Immune recovery after autologous stem cell transplantation is not different for HIV-infected versus HIV-uninfected patients with relapsed or refractory lymphoma. Clin Infect Dis 2010; 50:1672–9. 20450419 10.1086/652866

[27] Richman DD Wrin T Little SJ Petropoulos CJ Rapid evolution of the neutralizing antibody response to HIV type 1 infection. Proc Natl Acad Sci U S A 2003; 100:4144–9. 12644702 10.1073/pnas.0630530100 PMC153062

[28] Huang KH Bonsall D Katzourakis A B-cell depletion reveals a role for antibodies in the control of chronic HIV-1 infection. Nat Commun 2010; 1:102. 20981030 10.1038/ncomms1100 PMC2963804

[29] Salgado M Swanson MD Pohlmeyer CW HLA-B*57 elite suppressor and chronic progressor HIV-1 isolates replicate vigorously and cause CD4+ T cell depletion in humanized BLT mice. J Virol 2014; 88:3340–52. 24390323 10.1128/JVI.03380-13 PMC3957943

[30] Hutter G Zaia JA Allogeneic haematopoietic stem cell transplantation in patients with human immunodeficiency virus: the experiences of more than 25 years. Clin Exp Immunol 2011; 163:284–95. 21303358 10.1111/j.1365-2249.2010.04312.x PMC3048611

[31] Picker LJ Reed-Inderbitzin EF Hagen SI IL-15 induces CD4 effector memory T cell production and tissue emigration in nonhuman primates. J Clin Invest 2006; 116:1514–24. 16691294 10.1172/JCI27564 PMC1459071

[32] Wright JK Naidoo VL Brumme ZL Impact of HLA-B*81-associated mutations in HIV-1 Gag on viral replication capacity. J Virol 2012; 86:3193–9. 22238317 10.1128/JVI.06682-11 PMC3302318

[33] Li CR Greenberg PD Gilbert MJ Goodrich JM Riddell SR Recovery of HLA-restricted cytomegalovirus (CMV)-specific T-cell responses after allogeneic bone marrow transplant: correlation with CMV disease and effect of ganciclovir prophylaxis. Blood 1994; 83:1971–9. 8142663

[34] Rueff J Medinger M Heim D Passweg J Stern M Lymphocyte subset recovery and outcome after autologous hematopoietic stem cell transplantation for plasma cell myeloma. Biol Blood Marrow Transplant 2014; 20:896–9. 24631739 10.1016/j.bbmt.2014.03.007

[35] Deng K Pertea M Rongvaux A Broad CTL response is required to clear latent HIV-1 due to dominance of escape mutations. Nature 2015; 517:381–5. 25561180 10.1038/nature14053 PMC4406054

