# Supplementary material for: Proof-of-Principle for Immune Control of Global HIV-1 Reactivation In Vivo
Source: Clin Infect Dis. 2015 Mar 16;61(1):120–8. doi: 10.1093/cid/civ219 (PMC4463006; doi:10.1093/cid/civ219)
Supplement: Supplementary Data [file supp_61_1_120__index.html]

Proof-of-principle for immune control of global HIV-1 reactivation in vivo — Proof-of-Principle for Immune Control of Global HIV-1 Reactivation In Vivo — Proof-of-Principle for Immune Control of Global HIV-1 Reactivation In Vivo — Supplementary Data 

# Proof-of-Principle for Immune Control of Global HIV-1 Reactivation In Vivo

## Supplementary Data

Supplementary Data

**Files in this Data Supplement:**

- Supplementary Data - Docx file
- Supplementary Table 1 - docx file
