# Supplementary material for: Proof-of-Principle for Immune Control of Global HIV-1 Reactivation In Vivo
Source: Clin Infect Dis. 2015 Mar 16;61(1):120–8. doi: 10.1093/cid/civ219 (PMC4463006; doi:10.1093/cid/civ219)

**Supplemental clinical details**

A 59 year old Zambian male presented with a two month history of fatigue and weight loss. Bone marrow and blood tests were consistent with a diagnosis of IgG kappa multiple myeloma. He was refractory to first line oral chemotherapy and achieved a partial response following 4 cycles of intravenous bi-weekly bortezomib and dexamethasone. He received ESHAP (etoposide, methylprednisolone, cytarabine, cisplatin) and GCSF (granulocyte colony stimulating factor) to mobilize stem cells to the periphery after which 4 x 10^6^ cells/kg were harvested and frozen for subsequent transplantation. The autologous stem cell transplant (ASCT) also contained NK (0.18%), NKT (2.3%), CD4+ T cells (32.7%), CD8+ T cells (23.4%) and monocytes (1.5%). CD19+ B cells were not detected (Table 1).

At this time, screening uncovered HIV-1 seropositivity with a viral load (VL) < 50 copies/ml, a positive proviral HIV-1 DNA by qualitative real time PCR, a CD4+ count of >300/mm^3^ and infected with subtype C virus. The patient had been resident in the UK for 10 years during which time he had not experienced HIV-associated opportunistic infections. His HLA Class I type was HLA-A*29, *30, HLA-B*44, B*81:01, HLA-C*07, C*18. The HLA-B*81 allele is known to be enriched in African EC [1]. The patient did not possess known protective KIR alleles[2, 3]. The combination of undetectable VL, high CD4+ count, no opportunistic infections in a 10 year period and the HLA-B*81 allele were consistent with HIV-1 elite controller EC status.

**Supplementary Methods**

Calculation of transfer of HIV-1 specific CD8 T cells: Approximately, 19 billion total cells were transplanted of which 14.1% were memory CD8 T cells. 2.05% of cells were HIV-1 specific for TL9 and LY9 epitopes (the two strongest mapped epitopes) by intracellular cytokine staining (Figure 3). Assuming comparable cell viability at transplantation as for ICS experiments (47·4%), then the subject received ~26 million HIV-1 reactive CD8+ T cells.

CD8 T cell antiviral assay (extracellular p24) The assay was performed according to the protocol from Saez Cirion *et al* [4]. Briefly, 10^5^ CD4+ T-cells were infected with HIV-1 BaL virus and CD8 T-cells were added at 1:1 ratio at the same time as virus. Media alone was added to control wells and the assay was carried out in triplicate. After 3hrs cells were washed and cultured for 10 days in RPMI 1640 supplemented with 10% FBS, penicillin/streptomycin (100U/ml) and IL-2 (at 10pg/ml).

Intracellular cytokine staining PBMC were stimulated with 2μg/ml of peptide for 6 hrs in the presence of CoStim™ antibodies (BD Biosciences), protein transport inhibitors BFA and monensin, plus anti CD107a-PE antibody. Cells were stained with viability dye followed by CD4+, CD8+, CD27 and CD45RO+ antibodies. Cells were fixed and permeabilized (CytoFix/CytoPerm™ kit from BD Biosciences) then incubated with anti CD3, IFNγ, TNFα and IL-2. At least 4 x 10^5^ lymphocytes were acquired on a BD LSRII flow cytometer and analyzed using FlowJo (Tree Star Inc.), Pestle and Spice software (via Dr M. Roederer, NIH). Mock stimulation with 0.005% DMSO was performed as a negative control to assess background staining and results are shown following background subtraction.

Tetramer staining PBMC were incubated with PE labeled HLA-B*81 TPQDLNTML tetramer (in house) prior to staining with CD3, CD4+, CD8+, CD27, CD4+5RO, CD38 PD-1 and CD57 antibodies. At least 2 x 10^5^ lymphocytes were acquired on a BD LSRII flow cytometer.

Flow cytometry of lymphocyte cells:NK and B cells were assessed in two separate flow panels. Cryopreserved samples from day -41, +21, +42 and +472 post-transplantation were thawed, rested, washed and stained with viability dye followed by the following antibodies: 1) CD8+-PE, CD16-PerCP, CD56-V450, CCR7-Alexa647 and HLA-DR-APC or 2) CD20-PE, CD27-PerCPCy5.5, CD19-V450, IgD-APC and CD38-Alexa700. Cells were fixed and permeabilized (CytoFix/CytoPerm™, BD Biosciences) then incubated with anti CD3-PECy7 and Ki67-FITC. At least 3 x 10^5^ lymphocytes were acquired on a CyAn™ ADP Analyzer flow cytometer (Beckman Coulter). Fluorescence Minus One and unstained cells were used as controls for gating and analysis in FlowJo.

## Neutralizing antibody assay: A selection of SGA-derived gp160 fragments were cloned and expressed with the pSG3Δenv plasmid (NIH AIDS Research and Reference Reagent Program, USA, Cat. No.11051) to give pseudotyped viruses. In addition to the generated pcDNA3.1/V5-His-TOPO^®^ (Invitrogen)clones encoding autologous patient Envs, the ZM233M.PB6 *env* (SVPC9;[5]) were obtained through the NIH AIDS Research and Reference Reagent Program (USA), and the 93MW965.26 *env* clone was provided by D Montefiori (Duke University Medical Center, USA) through the Comprehensive Antibody Vaccine Immune Monitoring Consortium (CA-VIMC), as part of the Collaboration for AIDS Vaccine Discovery (CAVD). Virus neutralization assays were performed on TZM-bl cells as described previously [6]. Neutralization is measured as a reduction in Tat-induced firefly luciferase reporter gene expression after a single round of virus infection using 200 50% tissue culture infective doses (TCID_50_) of virus. Briefly, three-fold serial diluted complement-inactivated patient sera/plasma (from a 1:20 dilution) in DMEM supplemented with 10% FCS was incubated with virus for 1 hr at 37˚C in opaque 96-well cell culture plates. After this 10, 000 cells were added in DMEM supplemented with 10% FCS and dextran (Sigma-Aldrich, UK), to give a final DEAE concentration of 15 μg/ml. After 48 hrs incubation cells were lysed by addition of Bright-Glo luciferase reagent (Promega, UK) and luminescence read using a GloMax 96 Luminometer (Promega, UK). The 50% inhibitory concentration (IC_50_) was defined as the reciprocal serum dilution at which relative luminescence units (RLU) were reduced by 50% compared to virus control wells (average of eight replicates), following subtraction of background luciferase activity from cell control wells (average of eight replicates). The lowest serum concentration required to give 50% reduction in RLU (IC_50_) was determined by fitting the data to a sigmoidal equation using the XLFit 4 software (ID Business Solutions, Guildford, UK). Neutralization activity was assayed in duplicate and on at least two separate occasions, with average results being reported. Non-specific inhibition was assessed by testing all viruses against pooled human HIV-seronegative plasma (Sigma, UK), and the plasma samples against the Vesicular Stomatitis Virus (VSV) envelope (pseudotyped with pSG3∆env).

**Supplementary Text I: Cellular effects of chemotherapeutic agents used**

The subject received idarubicin (terminal half live 11.6 hr) and dexamethasone six months prior to the stem cell harvest, and Bortezomib (terminal half life 40 hr) /dexamethasone (terminal half life 4 hr) more than 2 months prior to stem cell harvest. Using a conservative estimate of 10 terminal half-lives these drugs would be fully cleared before HIV-1 rebound [7-10].

The subject received ESHAP (etoposide, methylprednisolone, cytarabine, cisplatin) therapy for 5 days and the GMCSF for 7 days prior to leukapheresis to harvest peripheral blood mononucleocytes including CD34+ cells mobilized from the bone marrow. Etoposide inhibits T cell proliferation and induces cell cycle arrest, and therefore could impact CD4 cell target numbers. Conservatively this drug has a terminal half-life of approximately 11 hours.  If this is used, along with a conservative estimate of 10 half-lives, then the drug would be fully cleared by 4-5 days[11].  Since there is no residual effect after drug removal, this means than any effect on cells would already have occurred by the time of harvest.  Both methylprednisolone which is a potent glucocorticoid with pleitropic effects on immune function and cytarabine, have terminal half-lives that are far less, at approximately 3 hours.  Therefore, there would be no drug left in cells by 30 hours post treatment [12, 13]. While the main effect of cytarabine is through DNA polymerase, there are some data demonstrating that cytarabine is incorporated into both DNA and RNA [14].  This has theoretical implications for the cells harvested, but the effect is difficult to determine. Given the strong expansion of both CD4+ and CD8+ T cells in our subject post-transplant, it does not appear that cytarabine had any residual effects on T cell proliferation.

Cisplatin which induces apoptosis has an elimination half-life of 43 min, and terminal half-life of 5.4 days[15].  25% of the drug is eliminated by 24 hours and by 5 days cellular concentrations are quite low. In one study, cisplatin was found as a reactive plasma ultrafiltrate species after 8 months of therapy[16].   This suggests that this compound could potentially have an impact on the cells harvested 8 days post ESHAP therapy.  But again, given the strong expansion of T cells post-transplant, there is little evidence that cisplatin limited the ability of the rebounding HIV-1 to propagate in CD4 T cells.

Overall, review of pharmacokinetic data of ESHAP drugs, suggests no to very little residual drug would have been retained within the cells that comprised the ASCT. Given that analysis of CD8+ T cells found they were strongly proliferative and were capable of producing cytokines and lytic molecules and HIV-1 reactivated and propagated in CD4 T cells which quickly reach numbers > 300 cells/µl, it does not appear any residual ESHAP produced deleterious effects in these cells that explained the virus rebound and subsequent control.

**Supplementary Text II: Mathematical modelling supports CD8+ mediated killing of infected CD4+ T cells**

Method: A standard viral dynamic model was analyzed in which infected cells in the eclipse phase, *I_1_*, transition to productive infection, *I_2_*, and with both populations as well as long-lived infected cells, *M**, being potential targets of immune cell killing. In the model cytolytic effectors, *E*, were stimulated by the presence of infected cells and grew with saturating kinetics. The model was represented as the following system of differential equations that were then solved numerically using Berkeley Madonna v8.3.18:

As the cells in the autologous transplant repopulate the T cell pool, the targets, *T*, are assumed to grow logistically at maximal rate *r* until they reach their carrying capacity *T_max_*. As T cell counts are observed to continue to drop during the first 6 days (Fig 1A) we assume T cells are lost at rate *d_T_* possibly due to residual melphalan in lymphoid tissues until time *t*_1_=6 d. To include this effect in the model, the term modeling this loss of T cells, *-d_T_T*, was multiplied by a Heavyside function, *H(t_1_-t)*, where *H(t_1_-t)* =1 when *t*<t_1_ and is 0 otherwise. Target cells, *T* and *M*, are infected at rates *β* and *β_M_*, respectively, where *M* represents a cell such as a macrophage or a less-activated CD4+ T cell that is long-lived once infected. As we are only interested in the early infection dynamics and these cells are inefficiently infected, for simplicity, we assume *M* is approximately constant as has been done in modeling the early response to antiretroviral therapy [17]. Infected cells in the eclipse phase, *I_1_*, transition into productively infected cells, *I_2_* at rate *k*, where *1/k* is the average length of the eclipse phase. Infected cells *I*_1_ and *I_2_* and long-lived infected cells, *M**, are killed by immune effector cells, *E*, with rate constants *δ_E1_*, *δ_E2_* and *δ_M_*.In addition, productively infected cells, *I_2_* and *M**, are killed by viral cytopathic effects at rates * *and *δ_M*_*, respectively. Productivelyinfected cells stimulate the immune effectors, *E*, which expand with a Michaelis-Menten or saturating growth rate described by the parameters *q* and *K*. We chose this density-dependent growth rate based on data obtained in vaccinated macaques where CD8+ T cell expansion slowed when antigen-specific CD8+ levels were raised prior to challenge [18]. Lastly, virus is assumed to be produced by productively infected cells, *I_2_* and *M**, at rates *p* and *p_M_*, respectively.

The important feature of the model is the inclusion of an HIV-specific effector cell response that is stimulated by HIV-infected cells. Before viral rebound the effector cell level is assumed to be very low. The response then builds up rapidly as the viral load rebounds (see blue curve in Figure 2) with the major expansion occurring near the viral load peak. The effector cells then control the viral infection bring the viral load down (green curve in Figure 2). The viral loads generated by the model agree with the ones measured in the patient (circles). The magnitude of the effector cell response is also similar in magnitude to the measured level of IFN-γ SFUs/million PBMCs shown in Figure 3.

The parameters in the model were chosen to be similar to those in the literature. The virion clearance rate *c* was set at *c*=23/day as estimated in Ramratnam et al [19]. The virion production rate from short-lived infected cells, *p*, was set at 20,000 virions per day, which is of the same order of magnitude as measured by Chen et al. [20]. The production rate from long-lived infected cells is not known and was set at 200 virions/day. The length of the eclipse phase is about one day [21, 22] and thus *k* was set 1 d^-1^ corresponding to a one day average eclipse phase. The death rate of short-lived infected cells by viral cytopathic effects, *δ*, was set at 0.9 d^-1^ so as to be somewhat smaller than the death rate from all causes of ~ 1.0 d^-1^ estimated by Markowitz et al.[23] The death rate of long-lived infected cells, *δ__*__ is not known and was set to 0.01 d^-1^ corresponding to a lifespan of 100 days, which is chosen somewhat shorter than that of uninfected naïve CD4 T cells [24]. The infection rate of CD4+ T cells, *β*, was set to 1.66 x 10^-8^ ml d^-1^, similar to the value estimated by Vaidya et al.[25] when all CD4+ T cells are assumed to be target cells. The infection rate of long-lived cells is not known and based on getting good agreement with the data was simply taken as 25% of the value of *β* he rate constants for effector cell killing of infected cells are not known and were chosen to get good agreement with the data. As ART is not being given, infection is ongoing and infected cell killing needs to be sufficient to prevent viral spread. At the viral load peak, the HIV-specific effector cell level is about 1000 cells/ml. With the killing rate constant of productively infected cells, *δ_ _*chosen as 0.001 ml d^-1^ productively infected cells are being killed by effectors at a rate of 1 d^-1^, similar to the rate of viral cytopathic death. The killing rate constant for infected long-lived cells, *δ_ _*was chosen 10-fold smaller as such cells probably express substantially fewer viral antigens. Interestingly, we found *δ_ _*chosen as 0.01 ml d^-1^ generated good agreement with the data.

Killing of cells in the eclipse phase has been previously suggested as being important in primate models. Another observation from the model was that the VL data were mainly explained by immune-mediated killing of productively infected cells and not killing of cells in the pre-integration ‘eclipse’ phase [26, 27]. When the model was limited to killing of cells within the eclipse phase only, the time of the virus peak changes and the entire viral load curve was decreased. Additionally, the rapid first phase VL decline as seen in this patient was not generated.

**References**

1. Emu B, Sinclair E, Hatano H, et al. HLA class I-restricted T-cell responses may contribute to the control of human immunodeficiency virus infection, but such responses are not always necessary for long-term virus control. Journal of virology **2008** Jun;82(11):5398-407.

2. Martin MP, Gao X, Lee JH, et al. Epistatic interaction between KIR3DS1 and HLA-B delays the progression to AIDS. Nat Genet **2002** Aug;31(4):429-34.

3. Bashirova AA, Thomas R, Carrington M. HLA/KIR restraint of HIV: surviving the fittest. Annu Rev Immunol **2011**;29:295-317.

4. Saez-Cirion A, Shin SY, Versmisse P, Barre-Sinoussi F, Pancino G. Ex vivo T cell-based HIV suppression assay to evaluate HIV-specific CD8+ T-cell responses. Nat Protoc **2010** Jun;5(6):1033-41.

5. Li M, Salazar-Gonzalez JF, Derdeyn CA, et al. Genetic and neutralization properties of subtype C human immunodeficiency virus type 1 molecular env clones from acute and early heterosexually acquired infections in Southern Africa. Journal of virology **2006** Dec;80(23):11776-90.

6. Montefiori DC, Karnasuta C, Huang Y, et al. Magnitude and breadth of the neutralizing antibody response in the RV144 and Vax003 HIV-1 vaccine efficacy trials. The Journal of infectious diseases **2012** Aug 1;206(3):431-41.

7. Voorhees PM, Dees EC, O'Neil B, Orlowski RZ. The proteasome as a target for cancer therapy. Clinical cancer research : an official journal of the American Association for Cancer Research **2003** Dec 15;9(17):6316-25.

8. Stewart DJ, Grewaal D, Green RM, et al. Bioavailability and pharmacology of oral idarubicin. Cancer chemotherapy and pharmacology **1991**;27(4):308-14.

9. Miyabo S, Nakamura T, Kuwazima S, Kishida S. A comparison of the bioavailability and potency of dexamethasone phosphate and sulphate in man. European journal of clinical pharmacology **1981**;20(4):277-82.

10. Schwartz R, Davidson T. Pharmacology, pharmacokinetics, and practical applications of bortezomib. Oncology **2004** Dec;18(14 Suppl 11):14-21.

11. Tranchand B, Amsellem C, Chatelut E, et al. A limited-sampling strategy for estimation of etoposide pharmacokinetics in cancer patients. Cancer chemotherapy and pharmacology **1999**;43(4):316-22.

12. Mollmann H, Rohdewald P, Barth J, Verho M, Derendorf H. Pharmacokinetics and dose linearity testing of methylprednisolone phosphate. Biopharmaceutics & drug disposition **1989** Sep-Oct;10(5):453-64.

13. Hande KR, Stein RS, McDonough DA, Greco FA, Wolff SN. Effects of high-dose cytarabine. Clinical pharmacology and therapeutics **1982** May;31(5):669-74.

14. Galmarini CM, Mackey JR, Dumontet C. Nucleoside analogues: mechanisms of drug resistance and reversal strategies. Leukemia **2001** Jun;15(6):875-90.

15. Vermorken JB, van der Vijgh WJ, Klein I, Hart AA, Gall HE, Pinedo HM. Pharmacokinetics of free and total platinum species after short-term infusion of cisplatin. Cancer treatment reports **1984** Mar;68(3):505-13.

16. Brouwers EE, Huitema AD, Beijnen JH, Schellens JH. Long-term platinum retention after treatment with cisplatin and oxaliplatin. BMC clinical pharmacology **2008**;8:7.

17. Perelson AS, Essunger P, Cao Y, et al. Decay characteristics of HIV-1-infected compartments during combination therapy. Nature **1997** May 8;387(6629):188-91.

18. Davenport MP, Zhang L, Bagchi A, et al. High-potency human immunodeficiency virus vaccination leads to delayed and reduced CD8+ T-cell expansion but improved virus control. Journal of virology **2005** Aug;79(15):10059-62.

19. Ramratnam B, Bonhoeffer S, Binley J, et al. Rapid production and clearance of HIV-1 and hepatitis C virus assessed by large volume plasma apheresis. Lancet **1999** Nov 20;354(9192):1782-5.

20. Chen HY, Di Mascio M, Perelson AS, Ho DD, Zhang L. Determination of virus burst size in vivo using a single-cycle SIV in rhesus macaques. Proceedings of the National Academy of Sciences of the United States of America **2007** Nov 27;104(48):19079-84.

21. Perelson AS, Neumann AU, Markowitz M, Leonard JM, Ho DD. HIV-1 dynamics in vivo: virion clearance rate, infected cell life-span, and viral generation time. Science **1996** Mar 15;271(5255):1582-6.

22. Dixit NM, Markowitz M, Ho DD, Perelson AS. Estimates of intracellular delay and average drug efficacy from viral load data of HIV-infected individuals under antiretroviral therapy. Antiviral therapy **2004** Apr;9(2):237-46.

23. Markowitz M, Louie M, Hurley A, et al. A novel antiviral intervention results in more accurate assessment of human immunodeficiency virus type 1 replication dynamics and T-cell decay in vivo. Journal of virology **2003** Apr;77(8):5037-8.

24. De Boer RJ, Perelson AS. Quantifying T lymphocyte turnover. Journal of theoretical biology **2013** Jun 21;327:45-87.

25. Vaidya NK, Rong L, Marconi VC, Kuritzkes DR, Deeks SG, Perelson AS. Treatment-mediated alterations in HIV fitness preserve CD4+ T cell counts but have minimal effects on viral load. PLoS computational biology **2010**;6(11):e1001012.

26. Klatt NR, Shudo E, Ortiz AM, et al. CD8+ lymphocytes control viral replication in SIVmac239-infected rhesus macaques without decreasing the lifespan of productively infected cells. PLoS pathogens **2010** Jan;6(1):e1000747.

27. Wong JK, Strain MC, Porrata R, et al. In vivo CD8+ T-cell suppression of siv viremia is not mediated by CTL clearance of productively infected cells. PLoS pathogens **2010** Jan;6(1):e1000748.

**Supplementary Figure 1: Clinical treatment for multiple myeloma and dynamic changes in neutrophil/lymphocyte proportions and T cell subsets over time.** Red - % neutrophils and green - % lymphocytes. Abbreviations: auto SCT: autologous stem cell transplant; ESHAP (etoposide, methylprednisolone, cytarabine, cisplatin); GCSF (granulocyte colony stimulating factor)


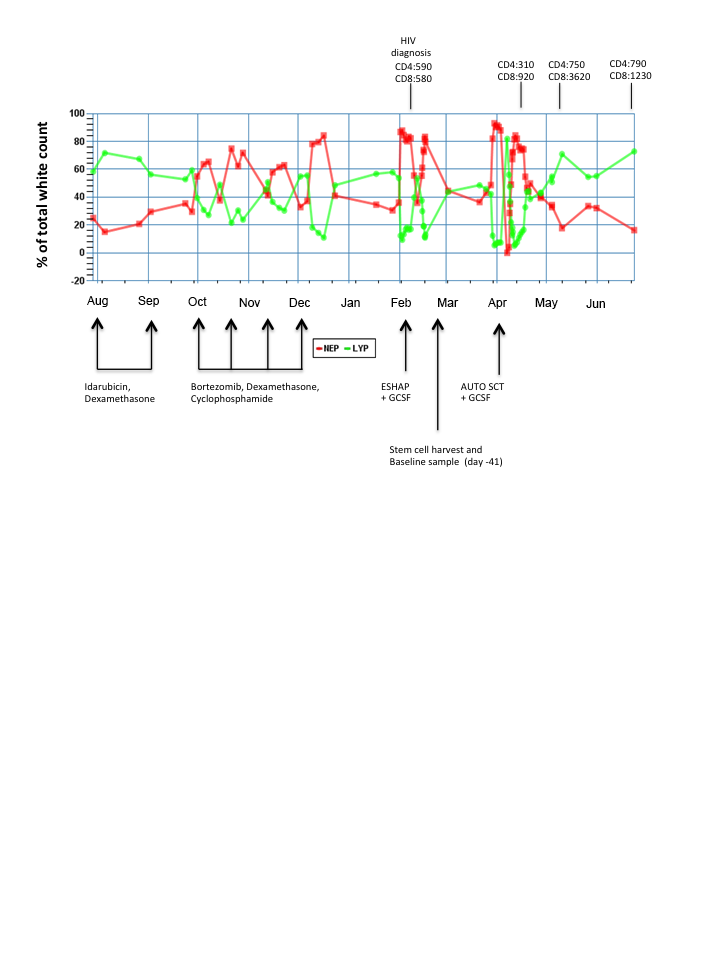

Supplement: Supplementary Data [file supp_civ219_civ219supp.docx]
