# Supplementary material for: Proof-of-Principle for Immune Control of Global HIV-1 Reactivation In Vivo
Source: Clin Infect Dis. 2015 Mar 16;61(1):120–8. doi: 10.1093/cid/civ219 (PMC4463006; doi:10.1093/cid/civ219)
Supplement: Supplementary Data [file supp_civ219_civ219supp_table1.docx]

Table 1: Neutralizing activity of sequential serum samples against autologous envelope was modest

| Day post-transplant | -360 | 6 | 12 | 15 | 63 | 76 | control ^b^ |
| --- | --- | --- | --- | --- | --- | --- | --- |
| *Autologous Env: ^c^* |  |  |  |  |  |  |  |
| A10 | <20^d^ | <20 | 129 | 114 | 52 | <20 | <20 |
| B9 | <20 | <20 | 54 | 91 | 30 | <20 | <20 |
| C1 | <20 | <20 | 158 | 158 | 29 | 23 | <20 |
| D2A | <20 | <20 | 104 | 138 | 56 | 21 | <20 |
| *Heterologous Env: ^e^* |  |  |  |  |  |  |  |
| ZM233M.PB6 | <20 | <20 | 82 | 96 | <20 | <20 | <20 |
| 93MW965.26 | 129 | 60 | 162 | 391 | 1488 | 1232 | <20 |
| VSV | <20 | <20 | 47 | <20 | <20 | <20 | <20 |

*^a^* Titres are expressed as the reciprocal dilution of serum required to reduce infectivity by ≥50% (IC_50_). *^b^* pooled Normal (HIV-seronegative) Human Sera. *^c^* The autologous *envs* were cloned from peak-vireamia (day +13). *^d^* <20, less than 50% reduction of infection was observed with the highest serum input assayed (1:20 dilution). *^e^* ZM233M.PB6, tier 2 clade C Env; 93MW965.26, tier 1 clade C Env; VSV, Vesicular Stomatitis Virus envelope glycoprotein.
